# What did we learn from preparing for cross-border transmission of Ebola virus disease into a complex humanitarian setting – The Republic of South Sudan?

**DOI:** 10.1186/s40249-020-00657-8

**Published:** 2020-04-21

**Authors:** Olushayo Oluseun Olu, Richard Lako, Joseph Francis Wamala, Patrick Otim Ramadan, Caroline Ryan, Ifeanyi Udenweze, Kibebu Berta, Argata Guracha Guyo, Alex Sokemawu, Michael Tukuru, Henry John Gray, Alex Chimbaru

**Affiliations:** 1 World Health Organization Ebola Virus Disease preparedness team, Juba, Republic of South Sudan; 2 National Ebola virus disease preparedness Incident Manager, Ministry of Health, Juba, Republic of South Sudan

**Keywords:** Lessons learnt, Ebola virus disease, Preparedness, Complex humanitarian settings, The Republic of South Sudan

## Abstract

**Background:**

Following the West Africa Ebola virus disease (EVD) outbreak (2013–2016), WHO developed a preparedness checklist for its member states. This checklist is currently being applied for the first time on a large and systematic scale to prepare for the cross border importation of the ongoing EVD outbreak in the Democratic Republic of Congo hence the need to document the lessons learnt from this experience. This is more pertinent considering the complex humanitarian context and weak health system under which some of the countries such as the Republic of South Sudan are implementing their EVD preparedness interventions.

**Main text:**

We identified four main lessons from the ongoing EVD preparedness efforts in the Republic South Sudan. First, EVD preparedness is possible in complex humanitarian settings such as the Republic of South Sudan by using a longer-term health system strengthening approach. Second, the Republic of South Sudan is at risk of both domestic and cross border transmission of EVD and several other infectious disease outbreaks hence the need for an integrated and sustainable approach to outbreak preparedness in the country. Third, a phased and well-prioritized approach is required for EVD preparedness in complex humanitarian settings given the costs associated with preparedness and the difficulties in the accurate prediction of outbreaks in such settings. Fourth, EVD preparedness in complex humanitarian settings is a massive undertaking that requires effective and decentralized coordination.

**Conclusion:**

Despite a very challenging context, the Republic of South Sudan made significant progress in its EVD preparedness drive demonstrating that it is possible to rapidly scale up preparedness efforts in complex humanitarian contexts if appropriate and context-specific approaches are used. Further research, systematic reviews and evaluation of the ongoing preparedness efforts are required to ensure comprehensive documentation and application of the lessons learnt for future EVD outbreak preparedness and response efforts.

## Background

The highly contagious and lethal nature of the Ebola virus disease (EVD) coupled with the negative impact that outbreaks have on the health system, social, cultural and economic development of affected communities ranks the disease as one of the most complex, dreaded and dramatic public health phenomena in recent time. Outbreaks of the disease have become more intense in Africa in terms of frequency, magnitude, duration and impact [1]. As of 18 February 2020, the most current and tenth outbreak of the disease in the Democratic Republic of Congo (DRC) had recorded 3432 cases and 2253 deaths making it the second-largest in the history of the disease [2]. The prolonged transmission of this outbreak is associated with challenges similar to those experienced in the West African outbreak (2013–2016): widespread community distrust and resistance due to strong traditional beliefs, preferences and lack of community engagement, chronic underdevelopment and weak health systems. These challenges are further complicated by insecurity due to ongoing armed conflict and recurrent attacks on response assets all of which continue to impede timely control of the outbreak [3–5].

The Republic of South Sudan (RSS) is considered to be at high risk of potential cross border importation from the current outbreak due to its proximity to the epicentres of the outbreak in North Kivu and Ituri provinces [6]. The Ministry of Health of RSS in collaboration with its partners has been implementing EVD preparedness interventions in seven high-risk states since August 2018 [7]. Three other neighbouring countries, Burundi, Rwanda and Uganda are also designated as high risk and are implementing similar EVD preparedness interventions.

The importance of instituting effective EVD outbreak preparedness measures for timely detection and containment of outbreaks cannot be overemphasized. The highly effective EVD alert system and preparedness measures instituted in one of the high-risk countries, Uganda ensured that it was able to timely detect and contain two cross border transmissions of the disease which were reported on 11 June and 29 August 2019 [8, 9]. This is similar to experiences from Nigeria in 2014 where an imported case was quickly identified and the resulting outbreak promptly contained [10]. Following the West Africa outbreak, the World Health Organization (WHO) developed an EVD preparedness checklist for use by its member states [11]. This checklist is being applied on a large and systematic scale for EVD preparedness for the first time in the four high-risk countries thus the need to review and document the lessons learnt from this experience. This is more pertinent given the complex humanitarian context and weak health system under which some of the countries such as RSS are implementing their EVD preparedness interventions.

This article highlights the progress made so far in the EVD preparedness efforts in RSS, the associated challenges, describes the key lessons learnt and proposes recommendations for using these to improve preparedness for the current and future outbreaks in the country and other complex humanitarian settings.

## Main text

### Setting the scene: the humanitarian context and EVD preparedness interventions and challenges in RSS

RSS is the World’s newest nation, emerging from an almost 50-year-long civil war with its northern neighbour to attain independence in 2011. Unfortunately, the new country soon plunged into a civil war in December 2013 which resulted in the internal and external displacement of over four million South Sudanese thus triggering a major humanitarian crisis. The renewed armed conflict in the country disrupted an already fragile healthcare system culminating in a severe shortage of healthcare workers, a disrupted supply management system for essential medicines and medical supplies, inadequate health financing, weak health governance and oversight system. These continue to constrain access to good quality, sustainable and affordable healthcare services in the country.

The health indicators in the country are poor; out of pocket spending on health is 54% while life expectancy at birth is 59 years [12]. The maternal mortality ratio is 789 per 100 000 live births while access to health care is estimated at 28% [13–15]. The government allocation to the health sector (less than 2%) is far below the Abuja target of 15% [16]. Malaria is one of the biggest causes of morbidity and mortality in the country; it is often complicated by malnutrition in children less than 5 years of age. Basic use of improved sanitation facilities and improved drinking water sources are estimated at 10 and 50% respectively while routine immunization coverage is 44% and consequently, outbreaks such as cholera and measles have been a major component of the ongoing humanitarian response in RSS [17–19].

Following the designation of the country as high risk for cross border EVD transmission in August 2018, the Ministry of Health and its partners instituted several EVD preparedness interventions. These include the designation of seven frontline states (Gbudwe, Jubek, Maridi, Tambura, Torit, Wau and Yei River) as high-risk, the establishment of a national EVD Incident Management System which comprise national and state coordination task forces, establishment of EVD surveillance at major points of entry, communities and health facilities in the high-risk states, activation and training of rapid response teams, establishment of laboratory capacity for diagnosis of EVD at the National Public Health Laboratory (NPHL), risk communication and preventive vaccination of 2974 frontline workers in the high-risk states.

Two external joint monitoring missions were conducted in November 2018 and March 2019 to monitor the country’s preparedness for EVD; the results showed improvement in the level of EVD preparedness in the country from 17% in November 2018 to 61% in March 2019 [20]. Aside from the risk of EVD, the country has also experienced recurrent outbreaks of measles, Yellow fever, Hepatitis E and cholera [21].

Despite these achievements, several challenges which continue to constrain timely implementation of EVD preparedness activities persist in the country. Table 1 highlights these challenges which include inaccessibility to many counties in the high-risk states due to insecurity and poor road infrastructure, weak health systems at the national and sub-national levels and weak coordination of the EVD preparedness efforts in the high-risk states.

**Table 1:** Challenges associated with Ebola virus disease preparedness in a complex humanitarian setting - Republic of South Sudan

Domain	Challenges	Proposed solutions
**Coordination**	Overlaps in the role of the incident management system and humanitarian health cluster	▪ Definition and delineation of Ebola virus disease (EVD) preparedness and response roles between the incident management system and health cluster. The cluster may assume the health partners’ coordination function of the incident management system. ▪ Regular orientation of health cluster partners on the incident management system and their respective roles and vice versa.
	Challenges of effective coordination of a large number of humanitarian partners	▪ Definition and consensus on the coordination structure as part of the preparedness plan and refining it through simulations. ▪ Strengthen public health emergency operation centers and leverage their capacities to streamline coordination at national and sub-national levels. ▪ Establishment of a national unified all-hazard multi-sectoral coordination platform. ▪ Effective mapping of partner capacities and comparative advantage to inform assignment of partners to relevant IMS functions. ▪ Flexibility to scale the incident management structure and functions to suit the scope and response needs of the outbreak. ▪ Definition and agreement among all partners on regional and national standards for training, simulations, personal protective equipment and EVD isolation units, etc. ▪ Conduct after-action reviews to learn from each response and improve coordination of preparedness and response.
**Programmes**	Weak health systems (inadequate human resources for health, weak information management system, disrupted supply chain management system, poor health financing, etc.)	▪ Integration of health system strengthening approach into the development and implementation of EVD preparedness plans. ▪ Definition and incorporation of strategies aimed at bridging the humanitarian-development nexus into EVD preparedness planning and response processes and interventions. ▪ Strengthening and use of other existing systems such as the NAPHS and integrated diseases surveillance and response system as platform for EVD and other outbreak preparedness and response.
	Community distrust and resistance to uptake of preparedness interventions	▪ Analysis of historical, political, cultural and social issues to understand factors and beliefs which influence community resistance to EVD interventions. ▪ Development and dissemination of tailored-made risk communication messages using existing community structures. ▪ Strong community engagement and participation in EVD preparedness and response interventions during the pre, intra and post epidemic phases. ▪ Regular assessments to rapidly identify and respond to changing communicaiton needs of the target populations.
**Operations**	Insecurity and attacks on response assets	▪ Integration of activities to advocate for and negotiate the security of EVD preparedness and response assets and access to EVD affected and high risk areas in EVD preparedness and response plans.
	Lack of access to high risk areas due to poor roads and insecurity	▪ Use of innovative approaches such as digital health technologies where feasible. ▪ Decentralization of preparedness and response interventions to high risk areas.
	High cost of operations due to humanitarian context (poor road infrastructure etc.)	▪ Phased approach to EVD preparedness through geographic and programmatic prioritization of EVD preparedness interventions. ▪ Continuous assessment and mapping of the EVD transmission risks to better classify high risk areas for targeted preparedness interventions. ▪ Engagement of relevant humanitarian clusters like the logistics cluster to prioritize outbreak prone locations for logistical support.

*IMS* Incident Management Systems, *NAPHS* National Action Plan for Health Security

### Reflections on the lessons learnt from preparing for EVD in RSS

Against the backdrop of the complex humanitarian setting, achievements and challenges highlighted in Table 1, implementation of the EVD preparedness interventions in RSS revealed several important lessons which we reviewed and synthesized into four main themes which are presented below.

#### Lesson 1: EVD preparedness is possible in complex humanitarian settings such as RSS by using a long-term health system strengthening approach

The appreciable improvements which were observed in the preparedness level for EVD in RSS despite a challenging environment have shown that effective EVD outbreak preparedness is feasible in complex humanitarian settings. This achievement is premised on some success factors which served as guiding principles in the implementation of EVD preparedness interventions. First, the preparedness efforts were based on the principles of bridging the humanitarian-development nexus thus emphasis was on a two-pronged approach. This approach prioritized the rapid establishment of critical outbreak response capacities that did not exist in the country previously while at the same time strengthening the health system capacity for longer-term management of disease outbreaks in general and EVD in particular.

For example, instead of depending on a regional referral laboratory (the Uganda Virus Research Institute in Entebbe) for confirmation of the Ebola virus using the reverse transcription polymerase chain reaction (RT-PCR) method, installation of an RT-PCR machine (originally procured for pandemic influenza surveillance), supporting infrastructure and training of national laboratory technologists to conduct the confirmatory test at the NPHL significantly improved the turnaround time for confirmation of suspected EVD samples in the second half of 2019 (unpublished WHO data). Aside from EVD, this machine is also used to test for other viral diseases such as influenza, Yellow fever, Marburg, Rift Valley fever, and other emerging pathogens. This capacity was rapidly adapted to test for the coronavirus disease 2019 (COVID-19) in early 2020. Likewise, the construction of semi-permanent points of entry screening points and installation of thermal scanners at Juba International Airport and Nimule land crossing border point within the EVD preparedness framework is contributing to screening for other diseases such as the newly detected COVID-19 and longer-term strengthening of the National Port Health System. Furthermore, the semi-permanent all-purpose infectious disease management unit which was originally constructed for EVD preparedness has been adapted and is being used for isolation of suspected and confirmed cases of COVID-19.

Second, development and implementation of innovative approaches including the use of appropriate technology were critical in identifying and addressing the key gaps in preparedness. For example, the development of a real-time and health system based integrated health facility supervision tool using digital health technology ensured timely identification of gaps in infection prevention and control and surveillance at the health facility level. This system provides valuable information for decision making for EVD preparedness to the state and national EVD taskforce teams as well as for the broader infection prevention and control in health facilities in the country.

Adapting this lesson for use in future EVD preparedness and response efforts requires the incorporation of strategies aimed at bridging the humanitarian-development nexus into EVD preparedness and planning processes in humanitarian settings right from the onset. This will ensure definition, prioritization and implementation of both immediate and long-term health system strengthening interventions for each preparedness and response pillar and phase.

#### Lesson 2: RSS is at risk of both domestic and cross border transmission of EVD and several other infectious diseases outbreaks hence the need for an integrated, sustainable and community-based approach to outbreak preparedness

Aside from the risk of cross border transmission of EVD from neighbouring countries such as the DRC and Uganda, the southern part of RSS sits in one of the ecological zones of the disease and jointly (with DRC) recorded the first-ever outbreak of EVD in 1976 [22]. The dual risk of an EVD outbreak (imported or emerging internally) alongside other epidemic-prone diseases such as cholera, measles, Yellow fever and COVID-2019 remain a plausible reality for RSS for the foreseeable future. Furthermore, the southern part of RSS which has the highest risk of cross border transmission of EVD from the current DRC outbreak has experienced several years of conflict, insecurity, and inadequate access to social services including health as a result of weak health systems and lack of access. These conditions mirror those of the epicentre of the current outbreak in Ituri and North Kivu provinces of the DRC hence the need to draw lessons which would prevent the occurrence of similar challenges should an outbreak occur in RSS.

Adoption of this lesson requires some critical actions. First, an integrated and sustainable approach to outbreak preparedness in the country which uses the National Action Plan for Health Security (NAPHS) as a platform to strengthen national capacity for surveillance, detection, diagnosis and management of not only EVD but all disease outbreaks is required [23]. Cross border exchange of preparedness and disease outbreak information, coordination and synchronization of preparedness and response interventions is also imperative. Second, strategic investment in intensive community engagement and participation is critical to gain the community trust long before an outbreak to prevent the type of community resistance which has played out in the DRC. As evidenced in the current outbreak in the DRC, the majority of EVD related deaths occur in the community (not in EVD treatment units) which indicates lack of community trust in government-run services in a complex political context. Similar perceptions are likely to exist in some areas in RSS. This further justifies the need for health system strengthening approach and a focus on building local capacity as a long-term preparedness strategy in epidemic-prone humanitarian settings. Additionally, a broader understanding of the historical, political, cultural and social contexts unique to each community is required to inform development and implementation of context-specific EVD preparedness programmes.

Third, in communities where trust has not been established and the context is volatile, negotiation of the security of EVD preparedness and response assets and access to EVD affected and high-risk areas should be integrated into and implemented as integral part of EVD preparedness and response interventions in all settings similar to RSS and the DRC.

Fourth, innovative approaches to overcome the logistic challenges of timely detection, investigation and response to potential outbreaks should be developed as early as possible. Such innovations may include the use of appropriate digital health technologies where feasible, decentralization of preparedness and response interventions and task shifting.

#### Lesson 3: phased and prioritized approaches are required for cost-effective EVD preparedness in complex humanitarian settings

From August 2018 to December 2019, an estimated USD 30.5 million was expended on EVD preparedness interventions in RSS [24]. This translates to an average of USD 2 million per month. While this is relatively low cost compared to the cost of outbreak response, it is a considerable amount for preparedness for one disease. This is more so since the country requires a total of USD 69 million to implement a five-year NAPHS which addresses all the International Health Regulation core capacities including preparedness for all the epidemic-prone diseases such as EVD and other zoonotic diseases including COVID-19.

The experiences during the ongoing preparedness efforts in RSS conveyed the challenges associated with predicting the location and timing of outbreaks. Unpublished data from the EVD alert management system showed that many of the alerts were detected outside the four locations where isolation units were sited. Transportation of patients from the site of an outbreak to any of these units would be logistically difficult in the complex humanitarian contexts like RSS. On the other hand, constructing a new EVD treatment centre in an epicentre would take a considerable number of days, during which the absence of proper isolation and infection prevention and control facilities could propagate the outbreak. This lesson calls for more cost-effective and all-hazard approaches to future EVD preparedness.

Addressing these lessons requires some key actions. First, evidence-based geographic and programmatic prioritization and phasing of EVD preparedness interventions are required. Each phase should have a clearly defined set of public health interventions. Such interventions should be prioritized according to those required nationwide and those required in the high-risk areas. For instance, preparedness interventions such as EVD risk communication and surveillance are required throughout the country during all phases of preparedness and response while interventions such as case management may be limited in geographic scope to specific locations where the risks of EVD importation are highest. The lessons learnt from neighbouring Uganda on their recent response experience to the importation of cases from the DRC to Kasese district conveyed an innovative approach used to overcome the challenge with predicting the location of a subsequent EVD confirmed case along their western border. A low-cost alternative (less than USD 30 000 per unit) similar to the CUBE (Chambre d’Urgence Biosécurisée, also called the Biosecure Emergency Room) which was developed by The Alliance for International Medical Action (ALIMA) [25] came in the form of rapidly deployable Ebola Isolation Units comprising of robust structures that could be stored centrally, distributed and assembled within one day. This is an innovative and practical approach that would be cost-effective and appropriate for use in the context of RSS where access remains a major challenge.

Second, continuous assessment and mapping of the EVD transmission risks would limit the number of interventions and partners required to implement them thus reducing costs.

#### Lesson 4: EVD preparedness in complex humanitarian settings is a massive undertaking that requires effective and decentralized coordination

The complexities and the large number of partners required to prepare for EVD transmission into complex humanitarian settings such as RSS is associated with some challenges. The meetings of the national EVD taskforce which was the strategic decision-making body were often long and inconclusive due to limited functionality of its technical working groups which should have discussed and resolved technical issues before presentation to the task force for approval. Inadequate guidance of the several partners most of who were clustered in the capital and one of the high-risk states, Gbudwe resulted in poor adherence to EVD norms and standards in the training of EVD staff and implementation of interventions. For instance, the inability of EVD partners to reach a consensus on the design, specifications and quantities of personal protective equipment, EVD isolation units, and content of EVD training modules and infection prevention and control standards and procedures often compromised the quality and timeliness of essential EVD preparedness interventions. Furthermore, the concentration of most implementing partners in the capital resulted in weak coordination in the high-risk states.

The introduction of two streams of coordination namely humanitarian and public health coordination addressed some of these challenges up to an extent. However, the new way of coordination came with its challenges. There was an initial lack of clarity about the allocation of tasks and responsibilities and linkage between the two streams of coordination. This was further complicated by duplication of roles and responsibilities in the national incident management system.

Addressing these coordination challenges requires a unified, strong and decentralized coordination platform for EVD preparedness in future. Such a platform should ensure that there is a generic architecture and terms of reference for various streams of EVD preparedness coordination and decentralization of the coordination mechanisms to the high-risk states and potential epicentres of outbreaks [26]. Furthermore, regional and national definition of standards in the areas of training, simulations, personal protective equipment and in the design and materials used for EVD isolation units and establishment of a central repository of reference materials and prequalified experts which countries like RSS can draw on is required.

## Conclusion

Despite a very complex humanitarian context, RSS made significant progress in its EVD preparedness efforts which demonstrate that EVD preparedness interventions can be rapidly scaled up in such contexts if appropriate and context-specific approaches are used. Despite the progress, the foregoing lessons show several gaps which should be addressed in the current and future preparedness efforts.

Moving forward, the gains made so far in the preparedness efforts for EVD and other outbreaks in RSS should be consolidated and used as an opportunity to build longer-term capacity for national and sub-national outbreak preparedness and response. In this regard, existing plans and systems such as the NAPHS, the Integrated Diseases Surveillance and Response System and the Early Warning, Alert and Response Network should be nurtured to maturity and used as sustainable, integrated and health system-based platforms for outbreak preparedness and response. This has become more pertinent given the emergence of new public health threats such as COVID-19.

Furthermore, the lessons highlighted above should be used as evidence to revise the WHO EVD preparedness guidelines. Lastly, further research, systematic reviews, monitoring and evaluation of the ongoing EVD preparedness efforts are required to ensure systematic documentation and application of the lessons learnt from previous and current outbreaks to future EVD preparedness and response efforts. The recently established Lancet Infectious Diseases Commission is a platform for implementing the foregoing recommendations [27].

## Data Availability

Not applicable.

## Abbreviations

ALIMA: The Alliance for International Medical Action
COVID-19: Coronavirus disease 2019
CUBE: Chambre d’Urgence Biosécurisée
EVD: Ebola virus disease
IMS: Incident Management Systems
NAPHS: National Action Plan for Health Security
RSS: Republic of South Sudan
RT-PCR: Reverse transcription polymerase chain reaction
WHO: World Health Organization
